# Effect of Shear on Pumped Capillary Foams

**DOI:** 10.1021/acs.iecr.3c00456

**Published:** 2023-05-01

**Authors:** Omotola Okesanjo, J. Carson Meredith, Sven Holger Behrens

**Affiliations:** † School of Chemical and Biomolecular Engineering, Georgia Institute of Technology, Atlanta, Georgia 30332, United States; ‡ Polymer Science & Materials Chemistry Practice, Exponent Inc., Atlanta, Georgia 30326, United States

## Abstract

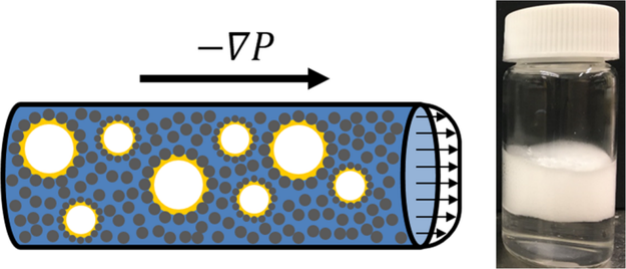

Foam flow in many applications, like firefighting and oil recovery, requires stable foams that can withstand the stress and aging that result from both shear and thermodynamic instability. Events of drainage and coarsening drive the collapse of foams and greatly affect foam efficacy in processes relying on foam transport. Recently, it was discovered that foams can be stabilized by the synergistic action of colloidal particles and a small amount of a water-immiscible liquid that mediates capillary forces. The so-called capillary foams contain gas bubbles that are coated by a thin oil-particle film and integrated in a network of oil-bridged particles; the present study explores how this unique architecture impacts the foams’ flow dynamics. We pumped capillary foams through millimeter-sized tubing (ID: 790 μm) at different flow rates and analyzed the influence of stress and aging on capillary foam stability. We find that the foams remain stable when pumped at higher flow rates but undergo phase separation when pumped at low flow rates. Our observations further show that the particle network is responsible for the observed stability in capillary foams and that network strength and stability of an existing foam can be increased by shearing.

## 1 Introduction

Liquid foams are gas–liquid mixtures found throughout products and processes in various industries. These multiphasic materials are versatile because their geometrical and rheological properties provide benefits such as high specific surface area, high viscosity, and finite yield stress.^1^ These benefits can be observed in applications such as froth flotation, where hydrophobic particles are separated from slurries through particle adsorption at the interface of rising bubbles; in firefighting, where foams are used to suppress combustion; in oil recovery where foams help to displace oil trapped in reservoirs; and in household/cosmetic products where foams are used as a delivery vehicle for active ingredients.^1,2^ In many of these processes, foams are pumped and experience shear stress; if the stress is below the yield stress, the foam can flow in a plug via slip at the wall. However, if the applied stress is above the yield stress, steady shear flow sets in, causing bubbles to slide past each other and liquid to be displaced from the Plateau borders and thin films.^3,4^ Foam performance in applications is determined by the stabilization mechanism as well as the interdependent dynamic processes of coarsening and drainage that govern the liquid fraction and bubbles sizes, which, in turn, affect the properties of the foam during use.^5^ A fundamental understanding of foam dynamics can therefore help in effectively engineering foam properties for different applications.^6^

Foams undergo changes from formation to complete collapse.^7^ Aqueous foams are formed by generating metastable gas bubbles in a continuous water phase; this process is governed by interfacial and chemical properties of the applied foaming agent and the technique used in foam generation.^8−11^ Once the foam is generated, the foam structure and composition evolve over time through the action of gravity that leads to liquid loss, surface tension that minimizes the gas–liquid interfacial area, and Laplace pressure differentials that drive diffusive coarsening, and inertial and viscous forces that can deform interfaces and hasten foam collapse.^12−14^ Coarsening and drainage are strongly coupled aging processes that induce internal dynamics affecting the rheological properties of foams.^12,15^ Drainage refers to the flow of liquid out of foams. In high-quality foams, where the gas volume fraction (ϕ) is above 70%, the liquid exists in the Plateau borders and thin films between the polyhedral-shaped gas bubbles of the foam. Drainage occurs under the influence of gravity and causes thinning of films separating bubbles, thus giving way to coalescence or bursting of bubbles. Capillary action counteracts drainage in foams and prevents complete loss of liquid from the foam. Diffusive coarsening is another means through which foams age; here bubble sizes evolve through a net diffusive flux of dissolved gas molecules across the liquid separating bubbles of different sizes. This flux causes larger bubbles to grow at the expense of smaller bubbles, which have higher Laplace pressure, leading to an increase in the average bubble size in the foam over time.^16^

**Figure 1:**
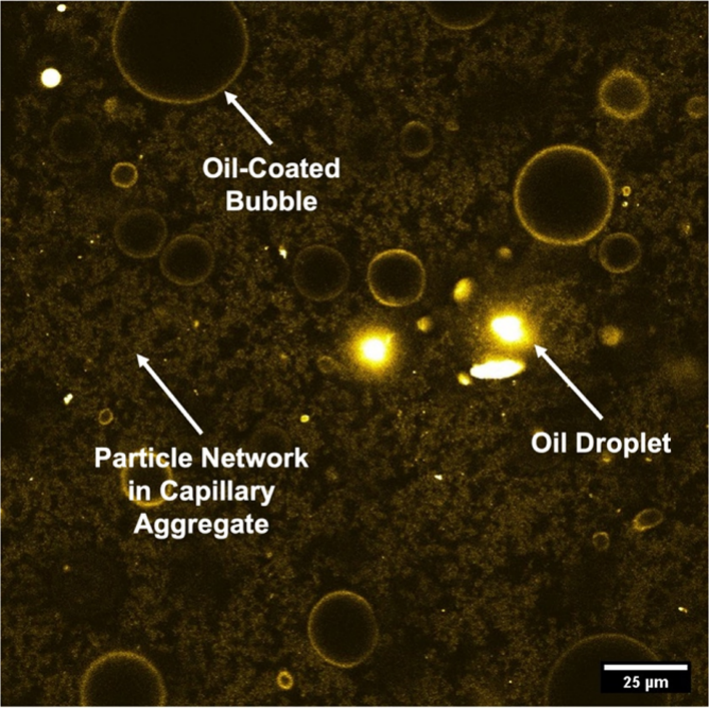
Confocal micrograph of a CF made from silica particles and fluorescently labeled TMPTMA oil. Bright yellow rings surrounding bubbles indicate the oily bubble coats; the diffuse coloring between bubbles stems from the oil bridges in the particle network, and the spots of intense brightness indicate droplets of oil. Whereas oil-coated bubbles and the network of oil-bridged particles are characteristic features of *all* CFs, the large oil droplets seen as very bright spots in the present image are not.

The rates of coarsening and drainage can be slowed when films between bubbles are thick and when particles accumulate in the Plateau borders, respectively.^13^ The high free energy of the gas–liquid interface can be reduced by surfactants that adsorb at the air–water interface, slowing down thinning and bursting of liquid films through steric or electrostatic repulsion of film surfaces and Marangoni flows that counteract surface tension gradients.^6,17^ Interfaces rigidified through the presence of particles can also arrest internal foam dynamics, allowing these gelled foams to remain stable for months. Colloidal particles in so-called Pickering foams have been shown to adsorb at the air–water interface, forming a densely packed layer that limits coarsening, and result in long-term foam stabilization.^18−21^ Another mechanism of foam stabilization, characteristic of the recently discovered capillary foams (CFs), combines the action of a small amount of a secondary immiscible liquid (oil) with interfacial particle adsorption to stabilize gas bubbles.^22,23^ CFs contain particle-stabilized, oil-coated bubbles embedded in a gelled ternary system of oil-bridged particles in water (capillary suspension).^24−26^ The confocal micrograph in Figure 1 shows this structure in CFs made from silica particles and trimethylolpropane trimethacrylate (TMPTMA) oil.^29^ CFs have been observed to remain stable for months after preparation.^23^

The formation and foamability of CFs have previously been characterized. It is understood that bubble stabilization occurs through particle-assisted spreading of oil around the air–water interface and is preceded by the initial formation of a network of capillary bridges between particles under mechanical agitation.^23^ The effects of the oil (type and concentration) and particle (wettability and concentration) on foamability and long-term stability have been qualitatively characterized.^23^ Fundamental rheological studies have shown that CFs, which have low gas volume fractions, can not only be extremely viscous but also have a yield stress comparable to that of surfactant foams (SFs) with high gas volume fractions.^27^

While rheological studies provide a foundation for understanding CF properties, for more practical purposes, it is useful to observe how CFs behave when they are pumped through cylindrical geometries, as they would be in many applications. For instance, the high viscosity and long-term stability of CFs suggest that they could potentially be useful as a displacing fluid in enhanced oil recovery (EOR) or CO_2_ sequestration where oil-stable foams are expected to improve sweep efficiency.^28^ Questions to be addressed for understanding the efficacy of CFs in applications involving flow include how gravity, capillary, viscous, and inertial forces influence the dynamics and stability. Here, we study the dynamics of CFs through visual observations of flow, analysis of aging behavior, and rheology measurements. CF flow through millimeter-sized tubing (ID: 790 μm) is visualized via microscopy and high-speed imaging. The observed behavior of these novel foams as a function of flow rate is explained and understood by considering the interdependent rheological and aging properties that influence foam flow. Lastly, we discuss how these results relate to the potential use of CFs in pore space applications such as EOR or sequestration.

## 2 Materials and Methods

### 2.1 Materials

Amorphous fumed silica particles modified with dichlorodimethylsilane and containing 50% residual SiOH on the surface according to the manufacturer were provided by Wacker-Chemie AG (Germany). Trimethylolpropane trimethacrylate (TMPTMA) and Rhodamine 6G (Fluka) were obtained from Sigma-Aldrich.

### 2.2 CF Preparation

Silica particles were first dispersed in methanol. The particle dispersion was centrifuged, and the methanol supernatant was removed. The particle sediment was rinsed by re-suspending the particles in deionized water, sonicating the solution to properly disperse the particles, and finally centrifuging the solution. The particles were rinsed at least five times to ensure that the methanol is effectively removed from the suspension. Particle sizes were determined by dynamic light scattering using a Malvern Zetasizer Nano ZS90. The hydrodynamic radius of the above silica particles in 5 mM NaCl solution at pH 4.9 is 403 nm. Silica particles used in this study are hydrophilic (θ_awp_ ∼ 30 ± 10) and have been shown to stabilize oil-in-water emulsions in an equal volume mixture of oil and water; it can therefore be concluded that these particles are wetted preferentially by the aqueous phase.^23,29^ Such particles are known to connect via capillary bridges in (bubble free) capillary suspensions and CFs.^24,29^

Silica particles were suspended in deionized water containing 5 mM NaCl at different volume fractions. TMPTMA was added to the silica suspension at 1 wt %. This UV-curable oil was used in the earliest studies of CFs,^22,30^ where scanning electron microscopy of cured and dried foams was employed to elucidate the architecture of CFs, and, while other types of oil have been explored as well,^22,23^ systems with TMPTMA as the oil phase have since become the most studied examples of CFs and were therefore selected for the present study as well. Rhodamine 6G dye was added to deionized water used to prepare CFs that were imaged under the microscope during pumping. Although some of the particles previously used to prepare CFs do not stabilize Pickering foams, the silica particles used here have also been reported to create Pickering foams in the absence of oil.^22^ We have, however, previously shown that the silica-stabilized Pickering foams have a lower modulus and yield stress than the CF produced in the presence of oil.^29^

CFs were prepared using a rotor-stator homogenizer (IKA Ultra-Turrax T10, rotor diameter of 6.1 mm, stator diameter 8 mm). CFs were prepared in a 10 mL BD syringe or 20 mL vial by mechanically frothing the oil/water/particle mixture with the IKA Ultra-Turrax at 30,000 rpm for 2 min. During frothing, the position of the mixer head is alternated between the surface and the bottom of the mixture at an interval of 20–30 s. At the onset of frothing, the homogenizer tip is placed at the surface of the oil/water/particle mixture to introduce air into the system. Subsequently, the homogenizer tip is submerged into the mixture to allow for high shear mixing of the particles and fluids. Densities of the silica/TMPTMA CFs used in this work range between 0.79 and 0.95 g/mL (at silica concentrations between 0.68 and 1.38 vol %)

### 2.3 Foam Flow Experiments

**Figure 2:**
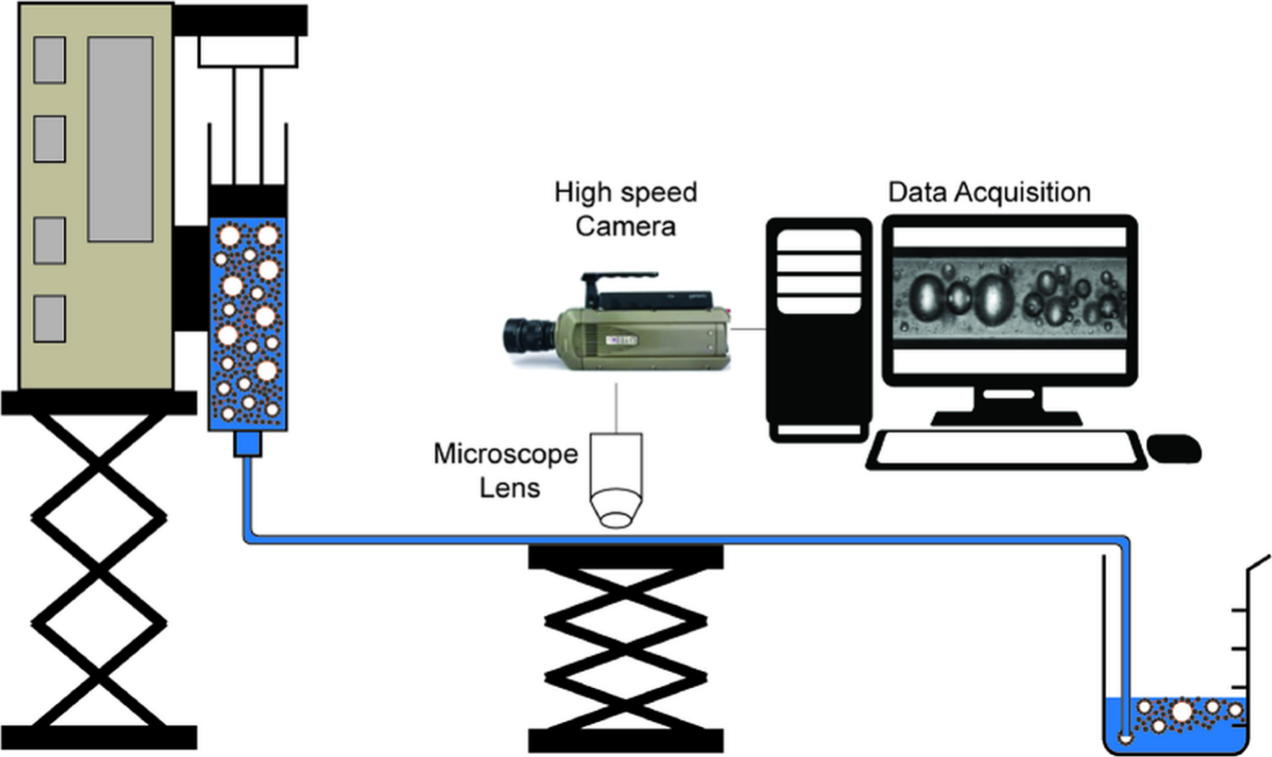
Schematic of the setup used in CF flow and flow imaging experiments (size proportions of the components not to scale).

CFs were prepared in a 10 mL BD syringe and were allowed to drain and settle for 30 min. The water at the bottom of the foam was first pushed out by displacing the water with the foam head, which is the more viscous fluid, and then the CFs were pumped through a polytetrafluoroethylene (PTFE) tube (ID: 0.79 mm; OD: 1.58 mm; L: 60 cm) into a collection vessel downstream using a KD Scientific (model 210) or a New Era (model 1010) syringe pump as shown in Figure 2.

Before each experiment, the tube was rinsed twice with 5 mL of deionized water, and the collection vessel was filled with 4 mL of water so that the foams did not dry out. CFs were pumped at flow rates between 0.1 and 10 mL/min. Foam flow is typically characterized by slip at the wall, and therefore all shear rates (γ̇) calculated and reported in this work are apparent.^29,30^ Shear rates reported for foam flow through the tube are determined by dividing the superficial velocity, obtained from the pump speed and cross-sectional area of the syringe and tube, by the tube internal diameter.

### 2.4 Foam Imaging Experiments

CFs were imaged using optical light microscopy on an inverted microscope (Nikon, Eclipse TE 2000-E, 10× objective). CF (pumped and unpumped) samples obtained from the middle portion of the foam head were placed in an observation cell, and multiple foam images were captured at different time points over a 24 h period. The average bubble diameter at each time point was obtained by averaging the diameters of at least 200 bubbles from multiple image frames. CFs prepared with Rhodamine 6G were pumped and imaged through the PTFE tubing on an upright microscope (Olympus, BX 51, 4× objective). A Phantom V7 high-speed camera was attached to the upright microscope to record foam flow at different flow rates. All microscopy images were processed using ImageJ software, while the video frames were analyzed with MATLAB.

### 2.5 Rheology Measurements

An Anton Paar MCR 501 rheometer equipped with a temperature controller was used to perform rheological measurements on CFs. To prevent inaccuracies that arise from shear localization and wall-slip in our rheological measurements, a vane geometry tool (diameter = 16 mm, length = 22 mm) was used in the yield stress measurements. A Couette geometry tool (bob diameter = 26.6 mm, bob length = 40 mm; cup diameter = 28.9 mm) was used to measure viscosity.

The CFs were prepared in the Couette cup of the rheometer. The temperature was maintained at 25 °C. Once prepared, the CFs were allowed to rest for 10 min before the start of the oscillatory and steady shear experiments. Frequency sweep oscillatory measurements within the linear viscoelastic regime were obtained before and after each experiment to monitor the elasticity of the foam.

## 3 Results and Discussion

### 3.1 Foam Flow

**Figure 3:**
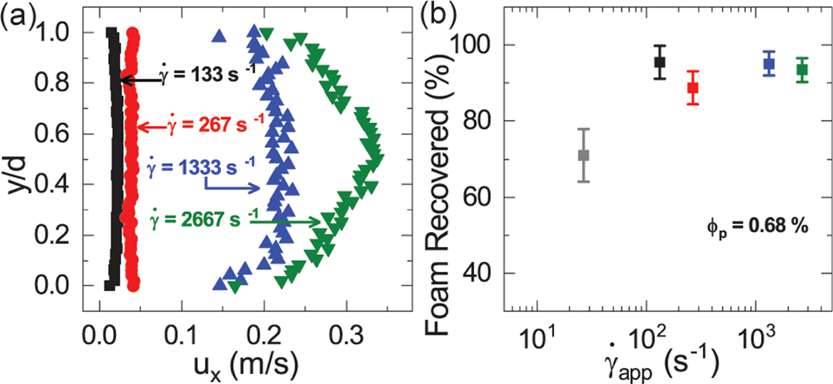
(a) Flow profile of CFs between . (b) CF volume recovered after pumping through the tube at various shear rates (γ̇). The gray square in (b) corresponds to unsteady flow at ∼26 s^–1^, while other squares represent flow rates for the profile shown in (a) in order of appearance from left to right.

CFs prepared at a particle volume fraction ϕ_p_ = 0.68 vol % (prior to frothing) and oil-particle ratio ϱ = 1.4 were pumped through a PTFE capillary tube to observe their stability and determine their characteristics at different flow rates. The particle network was labeled with Rhodamine dye to track bubbles, which appear darker than the oil-particle network under fluorescence excitation in microscopic imaging experiments (see Figure S1). The flow profile for CFs shown in Figure 3a was obtained by tracking CF bubbles in foam flow experiments with shear rates  s^–1^. The bubbles in the lowest shear rate experiment, when , were not tracked because the flow was unsteady in this case.

We observe from the video micrographs that CFs flow by slipping at the walls of the tube. At most flow rates, the foam maintains either bubble flow or slug flow in the tube. The foam flow profiles obtained in Figure 3a, with the exception of γ̇ ≈ 2667 s^–1^, confirm that the CFs maintain a near plug like flow profile that is typical of yield stress materials. At the lowest flow rate explored, when γ̇ ≈ 27 s^–1^, the flow starts out as slug flow but becomes more stratified toward the end of the experiment.^31^ The CF flow profile shows that significant shearing does not begin until , and the dynamic viscosity of the primary liquid begins to dominate. At , a plug regime exists in the center of the tube; however, the parabolic flow profile starts to develop at the edge of the tube, indicating light shearing. The flow profile for γ̇ ≈ 2667 s^–1^ shows complete shearing of the CFs and suggests that the foam yields throughout the flow cross section.

The volume of CF collected from the tubing was quantified and compared to the initial foam volume to determine how much of the CF was destroyed during the flow. The percentage of foam volume recovered, relative to the volume of foam prepared, is shown for flow at different shear rates in Figure 3b. The plot shows that CFs can be recovered from pressure-driven flow spanning 3 orders of magnitude of shear rate. We observe that for  s^–1^, 90% or more of the volume of CFs pumped are recovered on average. However, at a low shear rate of , we recovered a significantly smaller portion of the foam than at higher shear rates.

**Figure 4:**
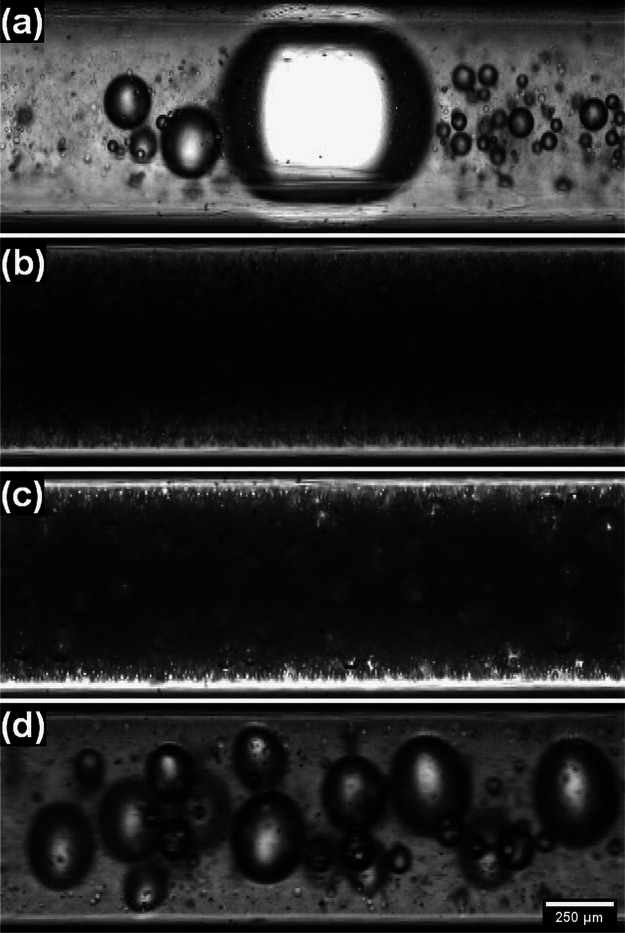
Micrographs of CFs flowing through the tubing at γ̇≈ (a) 26, (b) 133, (c) 266, and (d) 2667 s^–1^.

The uniformly high recovery of CFs at high and intermediate shear rates is surprising given the differences in the flow profile at these different shear rates. Microscopic images of CFs flowing at different rates are compared in Figure 4. At intermediate shear rates (see Figure 4b,c), we observe a large concentration of small- and medium-sized bubbles; additionally, we notice that the dense intact particle network present in the plug-like flow at these flow rates lowers the image contrast and makes the bubbles harder to see. On the other hand, we observe a mixture of large and small bubbles with higher image contrast at the lowest and highest shear rates (see Figure 4a,d, respectively).

The instability of the foam at the lowest pumping rate, inferable both from the bubble size distribution in Figure 4a and the reduced foam recovery (Figure 3b), is most likely related to the “jerky”, unsteady motion observed in this case and to the associated spatially and temporally localized shear that appears to compromise the foam integrity.

Figure 4d indicates that at the highest flow rate, the steady shear, present throughout the entire flow cross section according to the velocity profile (Figure 3a), also leaves a mark on the bubble size distribution, but in this case, the observed bubble growth is more limited, and foam recovery remains high (Figure 3a). We shall see below that exposure to steady shear in fact has a strengthening effect on the particle network in these CFs. To understand this better, it will prove useful to look at the short-term stability of static CFs before and after shear flow and at some rheological properties.

### 3.2 Short-Term Foam Stability

**Figure 5:**
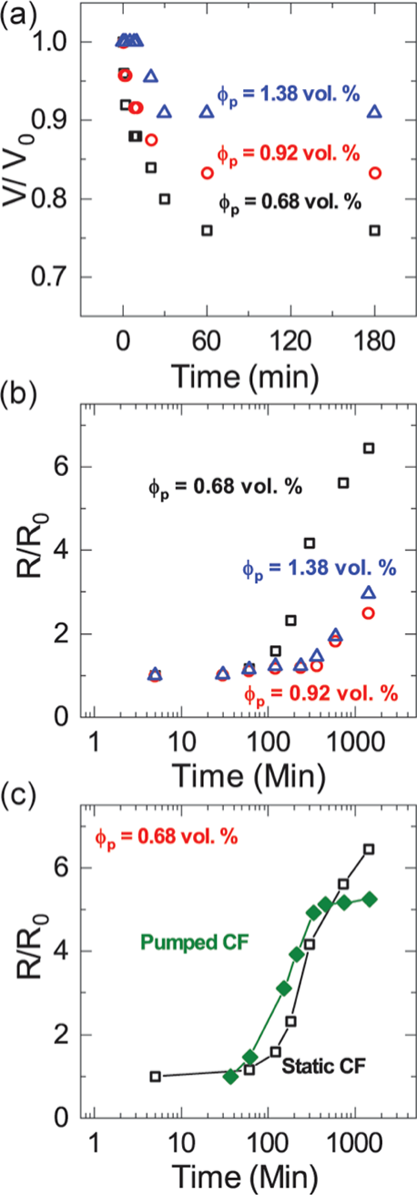
(a) Plot of relative foam volume change with time for static CFs at different particle volumes (ϕ_p_) and an oil fraction of 0.95 vol %. Silica/TMPTMA foams (black square, red circles, blue triangles). (b) Normalized average bubble size versus time for silica/TMPTMA static CFs at ϕ_p_ = 0.68% (black squares), ϕ_p_ = 0.92% (red circles), and ϕ_p_ = 1.38% (blue triangles). (c) Normalized average bubble size versus time for static CFs (black squares) and CFs pumped at γ̇ ≈ 1330 s^–1^ (green diamonds) when ϕ_p_ = 0.68%. Lines through data are guides to the eye.

We study the drainage and coarsening of static CFs in the initial hours after CFs are freshly prepared to understand CF aging through phase separation responsible for foam volume loss. We have quantified the short-term stability of silica/TMPTMA CFs against drainage by measuring the changes to the foam volume over time. Given that previous studies showed that the rheology and short-term stability of silica/TMPTMA CFs is strongly dependent on the particle volume and not the volume of the oil,^29^ we monitored the changes in the foam volumes of silica/TMPTMA CFs prepared at different particle volume fractions (ϕ_p_). We vary the silica particle concentration between 0.68, 0.92, and 1.38 vol % while keeping the TMPTMA oil concentration constant at 0.95 vol %. Figure 5a shows the relative change in foam volume over the experimental time for CFs at different particle volume fractions. The combined volume of the foam head and released water does not change in this time window, indicating that the reported changes in foam volume are due to gravitational drainage from the foam head and not due to loss of air from the bubbles.

The data in Figure 5a show that drainage occurs within the first hour after production of the silica CF and that the relative foam volume change is less pronounced the higher the particle volume fraction. As the density of particle network increases, more water is retained in the CF.

In classical SFs, drainage events are accompanied by diffusive coarsening, and these two processes characteristic of aging reinforce each other.^16^ The action of drainage thins the liquid barrier between bubbles and promotes diffusive coarsening; as smaller bubbles shrink, the rate of drainage also increases because there is more space for drainage to occur, which in turn shortens the diffusion path for coarsening, and so on.^15^ Coarsening in silica/TMPTMA CFs was quantified by measuring the increase in the average bubble sizes over a 24 h period beginning 5 min after the foam was prepared. The plots in Figure 5b show the normalized average bubble sizes over time for silica/TMPTMA CFs having the same particle volume fractions as those studied in the drainage experiments. We see a lag time in which bubble growth is insignificant, followed by a steady increase in bubble size over the remaining duration of the experiment, and observe that decreasing ϕ_p_ shortens the lag time and increases the subsequent rate of bubble growth. A strong similarity is apparent between the two coarsening curves for ϕ_p_ ≥ 0.92% (blue triangles and red circles in Figure 5b) and suggests that the coarsening behavior is insensitive to variations of ϕ_p_ at high network strengths.

The coarsening plot of CFs, though comparable to the coarsening behavior observed in SFs, displays differences worth noting. First, the time lag in bubble growth is longer for CFs; SFs exhibit a time lag on the order of minutes,^13,32^ whereas the time lag for CFs as observed in Figure 5b, is on the order of hours. The origin of this time lag can be attributed to a balance between drainage and diffusion in the foam. According to Magrabi,^33^ foam aging is controlled by drainage during the lag time and controlled by diffusion during the bubble growth regime. The lag time in CFs corresponds to long drainage times (∼1 h) observed in Figure 5a. In addition, we suspect that the composite layer of oil and particles surrounding the bubbles in CFs contributes to “weak coarsening”,^16^ which slows the drainage controlling the dynamics of the lag regime.

When the CFs are examined macroscopically over long periods of time, we observe that the foam becomes coarser and drier. In fact, after a period of ∼12 h, one can visually observe large bubbles in the foam matrix that could not previously be seen with the naked eye (see Figure S2). Bubble size analysis becomes more difficult and can be misleading after 24 h because the bubbles are difficult to observe with light microscopy.

Figure 5c shows that for a CF pumped at γ̇ ≈ 1330 s^–1^, bubble growth is observed as soon as pumping stops and the monitoring of coarsening begins about 32 min after the initial foam preparation. The average bubble size in pumped CFs plateaus ∼8 h (10^4^ s) after stopping the foam flow and is not observed to grow further within the duration of the experiment. We note that the value of the initial average bubble size for both static and pumped CFs are similar in these experiments. However, the data show that foam flow leads to a faster onset of bubble growth, *i.e.,* shorter lag time. This early bubble growth points to a disruption of the gel particle network between bubbles and/or a compromise of the oil-particle coating that stabilizes the bubbles in CFs. As Figure 4d suggests, high flow rates are capable both of breaking the particle network and of inducing bubble growth in CFs. It is plausible that after the stabilizing particle network has been compromised by the flow induced shear, drainage in the collected, relaxing foam is initially unimpeded and therefore very fast. Consequently, bubbles can grow without a significant delay until the stabilizing network has reformed, possibly stronger than before, with the temporary disruption and reorganization of the gel network having a strengthening (“annealing”) effect.

The drainage and coarsening experiments suggest that the silica/TMPTMA CFs undergo structural rearrangements initially before settling into a metastable state where foam aging kinetics are slower. An important point to note, however, is that the aging kinetics is dependent on both the particle network strength, which is a function of ϕ_p_, and the shear history of the foam. By increasing the particle volume fraction, the intermittent aging kinetics of the foam can be reduced because a stronger particle network better resists changes to the foam structure.^29^ In contrast, when the foam is subjected to higher shear rates, aging is accelerated because the particle network strength is compromised. The superior long-term stability of sheared CFs seems to indicate that sheared CFs may have a stronger gel network than unsheared CFs. To better understand how the network strength and shear history influence CF aging, we perform rheological measurements on the CFs used here.

### 3.3 Foam Rheology

By comparing the foam volumes (Figures 3b and 5a) and evolution of average bubble sizes of pumped and unpumped foams (Figure 5c), we have seen that pumping CFs through a tube influences CF aging. Further “before and after” pumping comparisons are called for but would require additional invasive handling of the pumped foam (such as foam transfer to a rheometer cell), which could compromise the foam. We therefore adopted a viable workaround: mimicking the shear conditions of tube flow in a Couette cell that can subsequently be used for the rheological characterization of foam properties related to the foam structure without additional handling. The Couette geometry, in which the foam is sandwiched between two smooth walls of concentric cylinders, also allows for wall slip in shear from motion relative to a smooth wall as is expected in tube flow.

**Figure 6:**
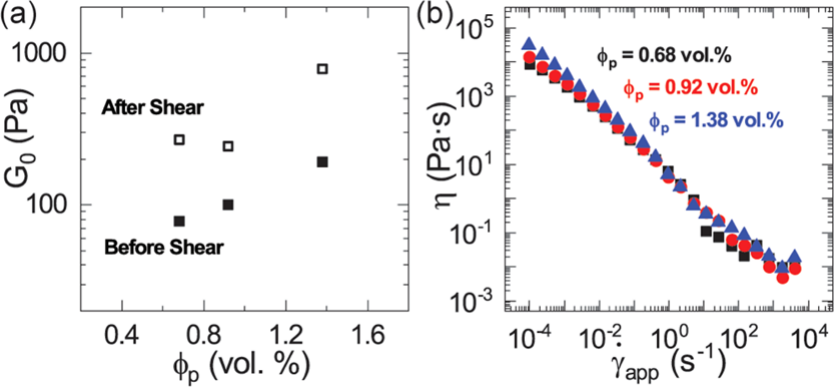
(a) Plot of the plateau modulus, *G*_0_, of CFs at different particle fractions, before (closed squares) and after (open squares) the controlled shear rate experiment. (b) Shear viscosity plots of CFs at ϕ_p_ = 0.68% (black squares), ϕ_p_ = 0.92% (red circles), and ϕ_p_ = 1.38% (blue triangles).

Silica/TMPTMA CFs were subjected to controlled shear rate (CSR) experiments in the Couette cell. Frequency sweep measurements were taken before and after the controlled shear rate experiments to determine the effect of shear on the CFs. The storage modulus (*G′*) of CFs at different particle volume fractions was measured in frequency sweep experiments, and we report the values of the plateau of the storage modulus (referred to as the “plateau modulus” *G*_0_ from here on) in Figure 6a; *G*_0_ is a measure of the foam elasticity. Comparing the initial and final moduli values of CFs subjected to the CSR, we observe an increase in the value of *G*_0_, which shows that a CF that has been subjected to shear has a higher modulus than an unsheared CF. The observation of higher moduli in sheared CFs indicates that the particle network within the CFs can be made stronger by the application of additional shearing (pumping) after the initial preparation of a CF.

The increased strength in the CF particle network induced by pumping is remarkable and suggests that there are changes that occur in the CF structure during shearing. It is likely that shearing of the CFs further drains the primary liquid from the foam and allows for higher connectivity of the particles by the secondary liquid. Additionally, we suspect that some of the bubbles are destroyed during shearing, and while their gas may escape, the oil and particles from their bubble coat may be incorporated into the particle network and strengthen it. The increase in the value of *G*_0_ is also consistent with the hypothesis that following the destruction or release of the bubbles from a CF, the surrounding particle network, which is a capillary suspension, should remain. Capillary suspensions usually have higher moduli than CFs (although the more commonly studied capillary suspensions are oil-continuous with water bridges connecting the particles instead of the other way around).^34,35^ We have previously shown that our CFs are less rigid than the water-based capillary suspensions that form their continuous phase.^27^

In Figure 6b, we plot the viscosity obtained for CFs at different particle volume fractions during the CSR experiment. The plot shows that CFs are shear-thinning. Although the viscosity decreases consistently throughout the plot, we note that the viscosity slightly increases at the highest shear rate accessible and suspect that this could perhaps be because of the formation of new bubbles at high shear rates. We observe that the shear viscosity plots of the CFs at different particle volume fractions collapse onto a single curve, indicating that the CF viscosity is insensitive to the particle volume fraction and the particle network strength in the explored ranges. The observation that the viscosity is independent of both *G*_0_ and ϕ_p_ suggests that the particle network is broken when yielding occurs and/or that the CFs flow by slipping on a thin film of fluid at the walls of the tool. At the end of the CSR experiments, we confirm that the CFs were indeed drained by noting that the bottom of the Couette cup contains mostly water while most of the foam is located at the edges of the Couette bob and cup where most of the shearing occurs.

The effects of shear on CFs lead to aging during and after the shearing process. We observe from rheology studies that pumping (or shearing) of the CFs increases *G*_0_ and further observe that coarsening in pumped CFs begins immediately after pumping plausibly because of increased drainage during shear. It is well known that pumping SFs accelerates foam aging and mostly destroys the foam; foam disintegration occurs because of diffusion-driven coarsening that leads to bubble growth and liquid drainage from the interfacial films.^10,36−38^ The particle network in CFs, however, reduces foam destruction during pumping and in the process, the network connectivity is increased. In a previous study, we have connected the increase in the foam modulus to aging observed in time sweep experiments.^27^

The combined results of our short-term stability and rheology studies not only provides us with better insight on CF flow dynamics but also provides understanding of how to manage and limit the effects of shear on CFs. To see this, we return to our observation of the CF pumped at low shear rates (γ̇ ≈ 27 s^–1^). We suspect that the loss of foam volume at the lowest shear rate is a result of aging (water drainage from the foam) accelerated by the effects of unsteady shear. At the low particle fraction, the gel particle strength is the weakest, and the CF does not retain water well (see Figure 5a). Although the foam is initially drained before pumping begins, drainage continues in the CF during the foam flow experiment. Continuous water drainage from the pumped foam makes the foam more susceptible to degradation under the applied stress and allows for the formation of large gas plugs. During the foam flow experiments at the lowest shear rate, we observed that the portion of the CFs closer to the syringe piston were not only drier but also coarser. When the foam closer to the piston passed through the tubing, it was completely separated into gas plugs and water suspension (see Figure S5). The gas bubbles separated from the foam escape when the foam is recollected at the tube exit and thus the foam volume loss is compounded.

**Figure 7:**
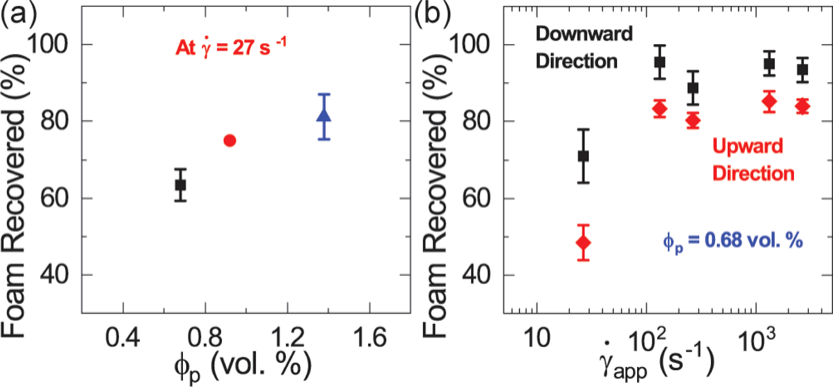
(a) Plot of CF recovered at different particle fractions when pumped (downward) at the lowest shear rate . (b) Plot comparing foam recovery when CF flow is directed downward (black squares) and upward (red diamonds).

To verify that phase separation through drainage and gas loss are indeed the cause of the foam volume loss, we investigated the effect of strengthening the gel particle network on foam flow at the lowest shear rate. In Figure 7a, we plot the percentage of foam recovered when CFs at different particle volume fractions are pumped at the lowest shear rate. We observe that by roughly doubling the particle volume fraction, from 0.68 to 1.38 vol %, the percentage of CFs recovered can be increased to 80%, an average increase by at least 15% from when the particle network strength is weaker.

The increase in CFs recovered with increasing particle volume fraction shows that the particle network helps not only to retain water but also to preserve the structure of the foam from phase separation during pumping. While we did not explore higher particle loadings, we expect that the effects of increasing the particle network strength on CF flow are only beneficial up to a certain point. Increasing the particle network strength beyond a certain point is bound to make flow through narrow tubes difficult and require impractically high pressures.

Lastly, we considered the stability of CFs when the flow is directed upward against gravity. Here, we invert the syringe pump setup and drained the CFs in an upward foam flow direction. We quantify, in Figure 7b, the relative foam volume recovered from the upward foam flow protocol and plot the comparative data for the downward foam flow protocol (Figure 3b). We see that the trends in the relative volume of CFs recovered with varying flow rates for the upward and downward flow protocol are similar. At any given flow rate, however, foam recovery is higher when the foam is pushed downward by the syringe piston than when it is pushed upward. We explain this behavior by looking at the nature of both fluids: Water and CFs. At low shear rates, the CF has very high viscosity and in fact behaves more like a rigid solid at low strains (see Figure S4b), whereas the viscosity of water is low by comparison and independent of strain rate. In the downward flow protocol, the displacing fluid is the foam, which easily displaces the less viscous water from the syringe and through the tube. On the other hand, in the upward flow protocol, drained water at the bottom of the syringe has to displace (“push”) the foam, a higher viscosity fluid. It is plausible that the CF is not uniformly displaced by the water in the upward flow protocol but is actually damaged by fingering channels of water that flow through the CF matrix. This behavior, known as viscous fingering, is usually observed between *two immiscible fluids* when the less viscous fluid is used to displace the more viscous fluid.^39,40^ Under the present conditions, we suspect that some of the water flows through the CF and by so doing causes bulk separation of the foam head and negatively impacts the bubbles and particle network in the CF. We show, in Figure S6, that particles dislodged from the CF can be seen settling at the top of the piston during the experiment when using an upward flow protocol. In addition to viscous fingering, CFs, and foams in general, can also be viewed as porous materials that can allow for flow of liquid through their matrices. Hence, water can flow through the foam and can alter the network structure that holds the foam together.

In summary, the results of our experiments prove that CFs can maintain various degrees of stability and rigidity under flow at small length scales (micro flows: <1 mm). The high viscosity and plug flow profile observed in the CF flow suggest that CFs could be useful as displacing fluids in foam flow applications like sclerotherapy,^38^ EOR,^41^ or soil remediation.^42^ Foam sclerotherapy, for example, involves flowing foam through varicose veins in the body to expel all the blood from them, ideally without mixing, *i.e.*, via plug flow. Uniform foam propagation would also be beneficial for foam flooding in oil recovery and soil remediation, as would good foam stability in contact with oil. Traditional foams typically do not afford that type of stability; in fact, oils are commonly used as antifoam agents.^43^ CFs, by contrast, are rather insensitive to contact with oils (see Figure S7), as one might expect, given the crucial role of an oily component in their stabilization mechanism. Still, more research and development will be necessary before CFs can be applied successfully toward any industrial goal.

## 4 Conclusions

We have investigated the dynamics of CFs at different flow rates in a small tube with a sub-millimeter diameter. Using light microscopy to observe the flow of CFs through the tube, we were able to trace the flow profile and understand how varying degrees of shear affect both the gel particle network and the oil-coated bubbles in CFs. We also show in this work how aging and foam rheology influence the volume of a CF recovered after the foam is pumped through tubing.

Our results show high recovery of CFs when the foams are pumped at intermediate and high shear rates and a lower foam recovery at the lowest shear rate explored where the foam flow becomes unsteady. Microscopy images and velocity profiles suggest that many of the bubbles are still entrapped by the unperturbed particle network at intermediate shear rates, whereas at higher shear rates, the particle network must rearrange, and larger bubbles can be observed. Aging studies show that the strength of the gel particle network is largely responsible for the observed dynamics of CFs. Aging in CFs can be controlled by tuning the strength of the gel particle network through varying the particle volume fraction, where higher particle volume fractions yield stronger gels. We also observe that pumping the foam through the tubing limits subsequent coarsening. This behavior is plausibly explained by the strengthening of the CF gel particle network when the foam is sheared. Frequency sweep and controlled shear rate experiments corroborate this hypothesis by showing that the elastic modulus of CFs increases after shearing. The observed strengthening of CFs by exposure to steady shear is a significant new finding because it is relevant to any application involving flow and further provides a novel way to tune the stability and mechanical properties of static foams.

The observed dynamics together with previous rheological studies on CFs suggests that CFs could be useful as displacing fluids for EOR and other applications, but more detailed feasibility studies are clearly needed.

## Supporting Information Available

Images of CFs bubbles under UV-light excitation, CF stability over time, amplitude sweep and viscosity measurements on CFs, CF flow images at low shear rate, and images of foam stability in the presence of crude oil (PDF)

## Notes

The material in this work is a part of the Ph.D. dissertation for Omotola Okesanjo that has previously been published here: http://hdl.handle.net/1853/66088.
