# Supplementary material for: Effect of Shear on Pumped Capillary Foams
Source: Ind Eng Chem Res. 2023 May 1;62(18):7031–9. doi: 10.1021/acs.iecr.3c00456 (PMC10178927; doi:10.1021/acs.iecr.3c00456)
Supplement: Supplementary file 1 — ie3c00456_si_001.pdf [file ie3c00456_si_001.pdf]

Supplementary Information

Effect of Shear on Pumped Capillary Foams

Omotola Okesanjo[1], J. Carson Meredith[1, *], and Sven Holger Behrens[1, 2, *]

[1]School of Chemical & Biomolecular Engineering, Georgia Institute of Technology, Atlanta, Georgia 30332, USA.

[2]Polymer Science & Materials Chemistry, Exponent Inc., Atlanta, Georgia 30326, USA.

*Email: sbehrens@exponent.com, carson.meredith@chbe.gatech.edu

Table of Content

1. Capillary foams bubbles under UV-light excitation Pg. 2
2. Capillary foam stability Pg. 2
3. Rheology of capillary foams Pg. 3
4. Capillary foam flow at low shear rate Pg. 5
5. Foam stability in presence of crude oil Pg. 6

1. Capillary Foam Bubbles Under UV Light Excitation

Capillary foams (CFs) were prepared from a suspension of silica particles (0.68 vol. %) containing Rhodamine 6G dye and Trimethylolpropane trimethacrylate (TMPTMA oil). Foam flow through the tubing was imaged using fluorescence excitation on an upright microscope. The particle network under excitation in **Fig. S1** appears grey because of the dye and the bubbles are dark.

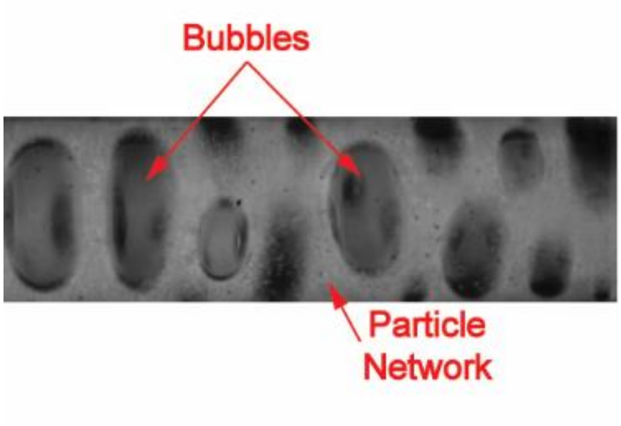

Figure S1: Image of capillary foam showing the bubbles enclosed within the particle network.

2. Capillary Foam Stability

CFs were observed to coarsening over time and bubbles can be seen in a 20 ml vial after 6 h.

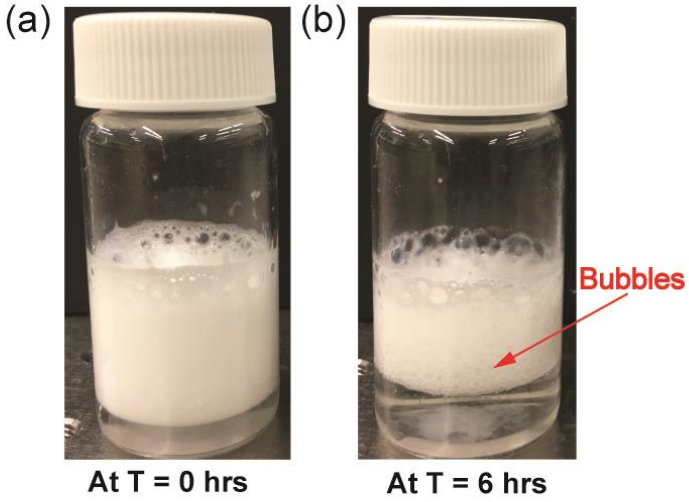

Figure S2: Images of bulk capillary foams (ϕ_p = 0.68 vol. %; ϱ = 1.4) showing foam evolution from a) when t = 0 h to b) when t = 6 h after preparation of the foam.

The bubble distribution of CFs was observed to increase with time as large bubbles grow and small bubbles shrink due to diffusive coarsening.

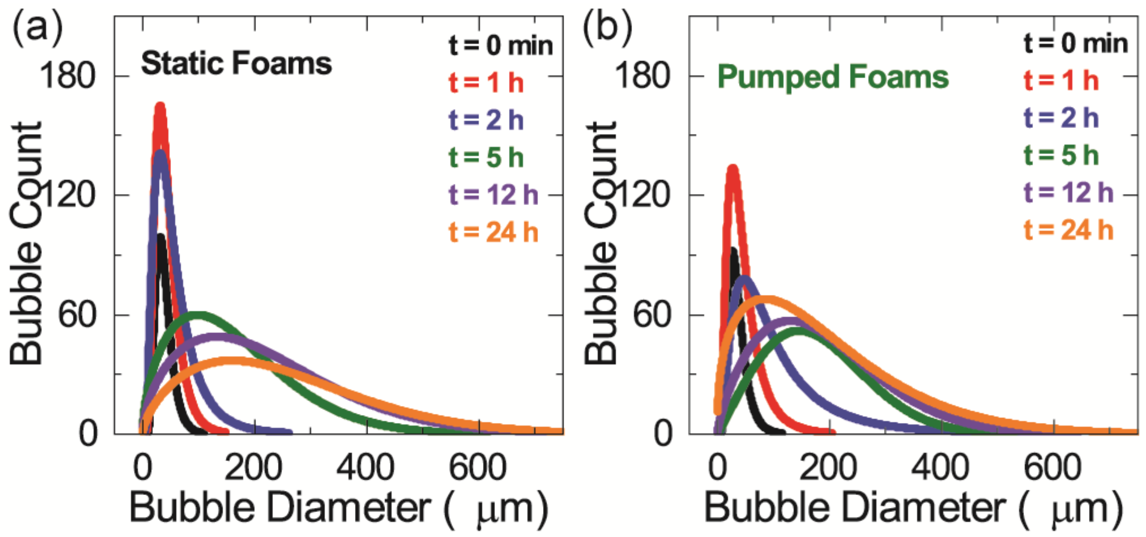

Figure S3: Plot of frequency distributions of bubble sizes in a) static and b) pumped capillary foams

3. Rheology of Capillary Foams

Silica/TMPTMA CFs are characterized at different particle volume fractions by measuring the storage modulus (*G'*) and the yield stress (σ_y) in rheology experiments. The values of the storage moduli and yield stress of the CFs are obtained by performing frequency sweep (FS) and controlled stress experiments (CSS), respectively, with a vane tool. We plot in **Fig. S4a** the values of the plateau of the storage modulus G_0 and σ_y at different particle volume fractions. Although the plot shows that the values of both σ_y and G_0 increase with increasing ϕ_p, at all particle fractions tested,

$G_0 > \sigma_y$. The higher rigidity in the particle network slows down the actions of drainage and coarsening in the CFs and thus explains the reduced aging effects in CFs observed at higher ϕ_p.

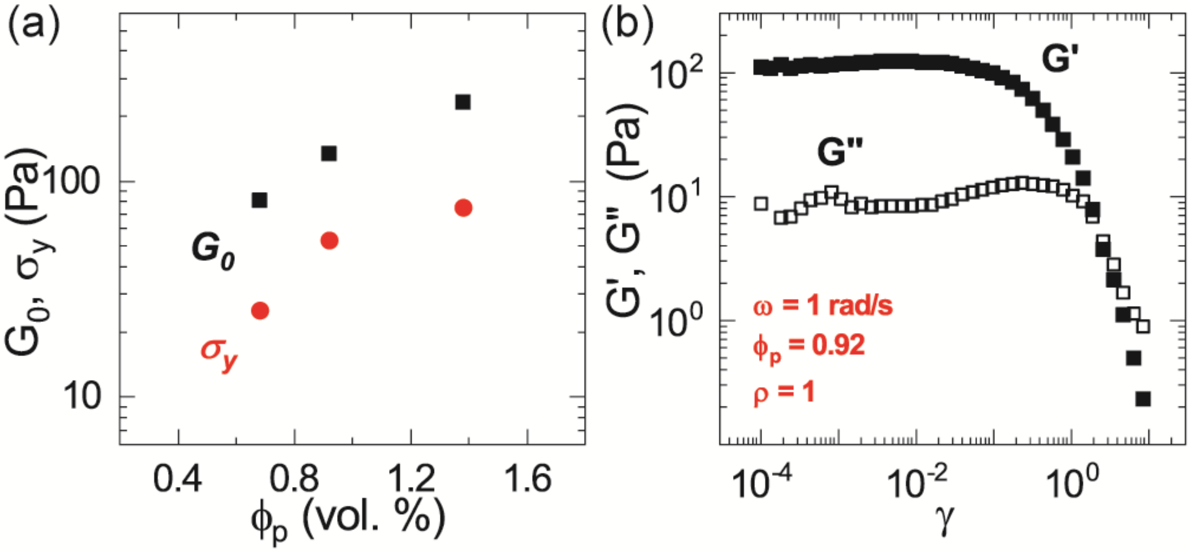

Figure S4: a) Plot of storage modulus *G* (black squares), and yield stress σ_y (red circles) of CFs at different particle fractions (ϕ_p). b) Plot of storage modulus (*G'*, closed squares) and loss modulus (*G''*, open squares) at different strains in an amplitude sweep experiment.

Amplitude sweep experiments under oscillatory tests were conducted on capillary foams prepared with silica particles at a volume fraction of 0.92 % and an oil-particle ratio of 1. **Fig. S4b** shows the raw storage and loss modulus plots obtained from the amplitude sweep experiments. The plot shows that *G'* is an order of magnitude higher than *G''* at low strains and indicates solid like behavior in CFs at low strains. At higher stains, when $\gamma \geq 0.1$, *G'* decreases and eventually falls below *G''* because the CF flows at higher strains.

4. Capillary Foam Flow at Low Shear Rate

Capillary foams pumped at a low shear rate were observed to undergo phase separation in the tube. The foam separated into plugs of water, gas and foam during pumping and the recovered foam was lower in volume than the foam volumes recovered at higher shear rates.

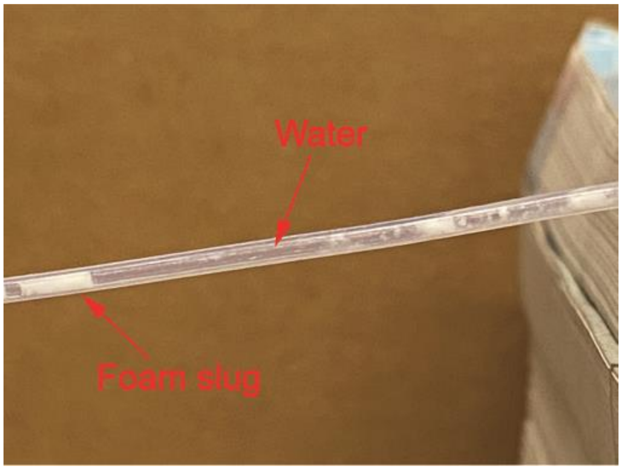

Figure S5: Image showing alternating flow of water and foam slug towards the end of capillary foam flow at the lowest shear rate ($\dot{\gamma} = 27\ \mathrm{s}^{-1}$).

Furthermore, we observed that when the capillary foam is pumped in an upward flow protocol as shown in **Fig. S6** below, water can flow through the foam, breakup the particle network and thus reduce the foam volume recovered downstream. The image in **Fig. S6** shows that the particle network dislodged from the foam head, as water flow through the foam, settles on the piston in the syringe.

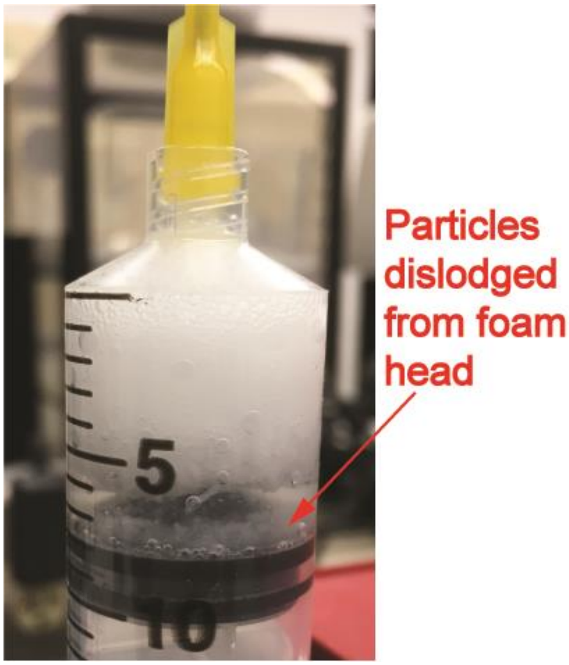

Figure S6: Image showing particles dislodged from capillary foam network settling at the top of the syringe piston.

5. Foam Stability in Presence of Crude Oil

Bulk foam stability in the presence of crude oil was evaluated by the addition of 0.8 ml of crude oil to surfactant foam (SF) and capillary foam. The surfactant foam was prepared by frothing a 2 vol. % suspension of sodium dodecyl sulfate (SDS) in a vortex mixer. The capillary foam was prepared by frothing a silica suspension (0.92 vol. %) containing 5 mM sodium chloride with TMPTMA at an oil-particle ratio of 0.5. **Fig. S7** shows a time series of images of both foams after the addition of crude oil. In surfactant foams, the initial volume of the foam head is halfed 10 mins after crude oil is added and continues to degrade with time. We observe that most of the foam head is gone about 2 h following the addition of crude oil. In CFs on the other hand, **Fig. S7** shows that the CF head remains stable in the presence of crude oil for at least 48 h after the addition of crude

oil. We suspect that the crude oil does not affect the CF because the CF is stabilized oil that bridges the particles in a strong network and coats the bubbles.

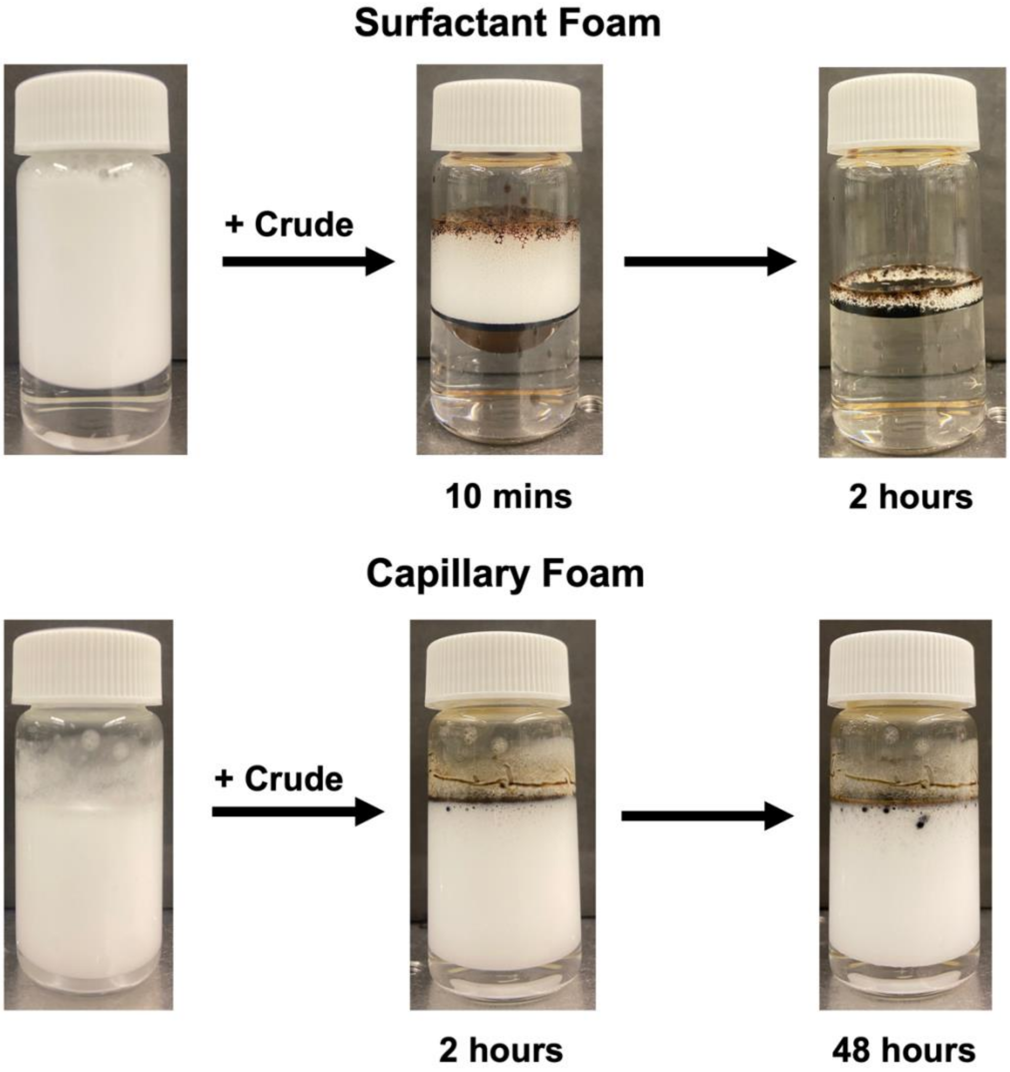

Figure S7: Time series showing surfactant foam (top) and capillary foam (bottom) stability after the addition of crude oil to the foam.
